# Assessment of the exposure to selected smoke constituents in adult smokers using in-market heated tobacco products: a randomized, controlled study

**DOI:** 10.1038/s41598-022-22997-1

**Published:** 2022-10-28

**Authors:** Dai Yuki, Akira Kikuchi, Takuya Suzuki, Chikako Sakaguchi, Danting Huangfu, Yasufumi Nagata, Aoi Kakehi

**Affiliations:** Scientific and Regulatory Affairs, Japan Tobacco Inc., 4-1-1 Toranomon Minato-ku, Tokyo, 105-6927 Japan

**Keywords:** Biochemistry, Biomarkers

## Abstract

The objectives of this clinical study were to demonstrate a reduction in exposure to selected harmful and potentially harmful constituents (HPHCs) in Japanese healthy adult smokers who switched to four in-market heated tobacco products. Eighty-nine smokers were randomly assigned for five days to one of six study groups: four groups who switched to one of the commercially available heated tobacco products; a group who continued to smoke their own brand of combustible cigarettes (CC); or a group who stopped smoking (SS). Fifteen biomarkers of exposure (BoE) to 14 HPHCs and pyrene were measured at baseline, Day 3 and Day 5 in 24 h urine and breath, under clinical confinement. Product consumption, nicotine uptake and subjective effects were also measured before and after product switching. On Day 5, significant reductions in most BoE relative to the CC group were observed after switching to heated tobacco products. No changes in BoE were observed between baseline and Day 5 in the CC group. Significantly, the magnitude of the reduction in exposure to most of the selected HPHCs observed in the heated tobacco product groups was close to that observed in the SS group.

## Introduction

Cigarette smoking is associated with increased risk of pulmonary diseases like chronic obstructive pulmonary disease (COPD), cardiovascular diseases (CVD), and cancers in a variety of organs^1^. It is reported that such smoking-related diseases are caused due to long-term exposure to a number of toxicants which are present in cigarette smoke following the combustion of tobacco in the cigarette^1,2^.

In 2001, the National Academy of Medicine (formerly known as Institute of Medicine) reviewed the scientific basis for tobacco harm reduction and came to the conclusion that reducing the risk of disease by reducing human exposure to tobacco toxicants is a feasible goal^2^. In 2012, the U.S. Food and Drug Administration (FDA) issued draft guidance on regulatory applications for a modified risk tobacco product (MRTP) defined as any tobacco product that is sold or distributed for use to reduce harm or the risk of particular diseases related to marketed tobacco products^3^. For MRTP applications, the FDA requires scientific evidence showing actual risk reduction, such as long-term epidemiology data, before it will issue an order of “risk modification.” In addition, there is an FDA requirement for issuing an order of “exposure modification,” namely, if scientific evidence demonstrates that exposure reduction and risk reduction can reasonably be expected without direct evidence of risk reduction. More recently, the FDA authorized a manufacturer to market a specific heated tobacco product as MRTPs and issued an order of “exposure modification” that is expected to benefit the public health^3,4^. Heated tobacco products are relatively new and have not been in available in the market for a long enough time period to gather epidemiological evidence regarding reduced risk of such products. Therefore, scientific evidence that shows reduction of human exposure to tobacco toxicants is extremely important to gain early insight into the potential for reduced risk.

In Japan, the country where there is now a high level of acceptance of heated tobacco products among current tobacco product users^5,6^, there are primarily two heated tobacco product systems on the market: (a) Indirect tobacco heating system (e.g., Ploom TECH/NTV), which indirectly heats tobacco with an aerosol produced from an electrical heating device; (b) Direct tobacco heating system (e.g., glo/THP and IQOS/THS), which directly heats tobacco with an electrical tobacco heating device. The results of chemical analysis of the aerosols generated by such heated tobacco products consistently showed that most of the measured selected cigarette smoke constituents were significantly lower than those found in combustible cigarettes (CC)^7–9^.

During the last decade, many clinical studies for some of the developed and marketed heated tobacco products have been conducted and reported a sustained reduction in human exposure to selected harmful and potentially harmful constituents (HPHCs^10,11^) among those who switched away from CC to some kind of heated tobacco product^12–18^. However, these studies cover only a portion of the currently marketed heated tobacco products. New and updated versions of heated tobacco products have been introduced annually in Japan, and continuous research is needed on these currently marketed products. In addition, as heated tobacco products become more numerous and diverse, comprehensive research is also important to investigate different products under the same conditions.

**Figure 1:**
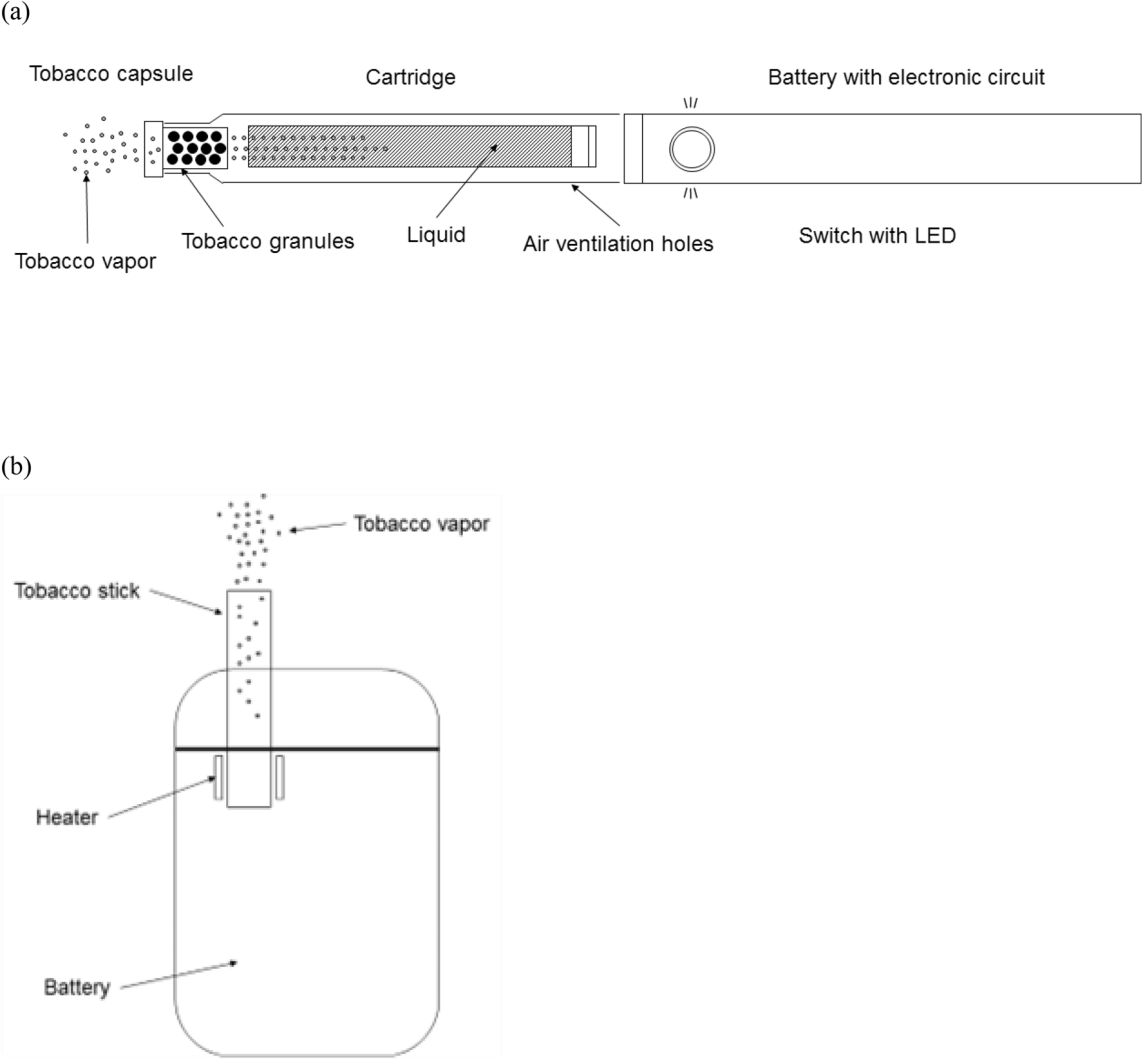
Schematic of two novel heated tobacco products: (**a**) IT2.0a and (**b**) DT2.2a.

In 2019 and 2020, two types of novel heated tobacco products (the indirect tobacco heating system platform 2 generation 0 version a [IT2.0a] and the direct tobacco heating system platform 2 generation 2 version a [DT2.2a]) have been developed and introduced by Japan Tobacco Inc. (Fig. 1). The chemical analysis of aerosols generated from the IT2.0a and DT2.2a showed that most of the measured selected cigarette smoke constituents were below quantifiable levels^19^. In addition, IT2.0a and DT2.2a barely registered a response when investigated for mutagenic and cytotoxic effects relative to the University of Kentucky Reference Cigarette (1R6F) in in vitro toxicological assays^19^. Such results would suggest that switching from CC to IT2.0a and DT2.2a examined in the present study reduces exposure to cigarette smoke constituents. Furthermore, the present study comprehensively investigated the reduction in human exposure to cigarette smoke constituents from IT2.0a, DT2.2a and two major in-market heated tobacco products (THP and THS).

The primary objective of the present study was to evaluate biomarkers of exposure (BoE) for selected HPHCs in the breath and urine of healthy adult smokers who either continued smoking CC, stopped smoking, or switched to using one of four in-market heated tobacco products (IT2.0a, DT2.2a, THP, and THS) over a 5-day period.

## Methods

### Study design

This was a randomized, controlled, six-arm parallel group, open-label, confinement study carried out at two clinics in Fukuoka, Japan. The study was conducted in accordance with Good Clinical Practice and the Declaration of Helsinki. Prior to the start of study, the study was approved by Hakata Clinic Institutional Review Board and registered at the UMIN Clinical Trials Registry (UMIN000041539, registered on 25/Aug/2020). All participants were recruited from the clinic-managed volunteer panels and provided written informed consent to participate in the study.

A study design schematic is shown in Table 1. Eligible adult smokers checked into the clinics in the evening of Day − 2 and were randomly assigned at a similar gender ratio on Day − 1 into one of the six study groups (a group who switched to an IT2.0a, a group who switched to a DT2.2a, a group who switched to a THP, a group who switched to a THS, a group who continued to smoke their own brand of CC, and a group who stopped smoking [SS]) after the inclusion/exclusion criteria had been assessed. Subjects were randomly assigned to one of six parallel study groups in equal ratio, and the randomization was stratified by the sites, gender and visiting cohorts in order to be well-balanced. Subjects were randomly assigned to one of six study groups using the electric data capture system and the investigators and site staffs were blinded to the randomization scheme.

**Table 1:** Study design schematic.

Day − 28 to Day − 3	Pre-observational period		Investigational period					Post-observational period
	Day − 2	Day − 1	Day 1	Day 2	Day 3	Day 4	Day 5	Day 6
Screening	Check-in	Baseline, continuing to smoke CCs	IT2.0a group: switch from CC to IT2.0a					Discharge
			DT2.2a group: switch from CC to DT2.2a					
			THP group: switch from CC to THP					
			THS group: switch from CC to THS					
			CC group: continue to smoke CC					
			SS group SS: stop smoking					

*CC* combustible cigarettes, *DT2.2a* direct tobacco heating system platform 2 generation 2 version a, *IT2.0* indirect tobacco heating system platform 2, *SS* stop smoking, *THP* tobacco heating product, *THS* tobacco heating system.

On Day − 1, subjects smoked their own brands of CC ad libitum within ± 10% of their self-reported daily consumption. From Day 1 to Day 5, all investigational tobacco products were recorded and dispensed by the site staff one-by-one, and the subjects were allowed to use their assigned tobacco product from 9:00 a.m. to 10:30 p.m. in supervised smoking areas designated separately between CC and heated tobacco products. It was set to allow subjects to use their assigned product for as longest as the site could secure. Subjects in the IT2.0a, DT2.2a, THP and THS groups used their assigned heated tobacco products ad libitum and were denied access to smoking areas designated for CC. Subjects in the CC group continued to smoke their own brands of CC ad libitum within ± 10% of their self-reported daily consumption. Subjects in the SS group abstained from smoking and were denied access to smoking areas. During clinical confinement, all subjects were provided with the same dietary menu without grilled or smoked foods to avoid exposure to some of the selected measured constituents from other sources. On Day 6, subjects were discharged after undergoing safety assessment procedures.

### Subjects

Adult healthy male and female Japanese smokers aged 21–65 years were eligible to participate if they smoked an average of 11 or more commercially available non-menthol CCs per day at screening, had smoked for at least 12 months before screening, and had a positive result for a urinary cotinine test (One Step Cotinine Test Device, Accuracy-One Inc., California, USA, cut-off concentration = 200 ng/mL). Before enrollment, the health status of all participants was confirmed by physical examination, medical history, vital signs, electrocardiogram and clinical laboratory testing. Main exclusion criteria were pregnant or breastfeeding females; males having a body mass index (BMI) of less than 18.5 or greater than 27.7 kg/m^2^ and females having a BMI of less than 16.8 or greater than 26.1 kg/m^2^; participants who had donated ≥ 200 mL of blood within 2 weeks or donated ≥ 400 mL of blood within 12 weeks (males) or 16 weeks (females) prior to Day 1 and participants who had any clinically relevant abnormal findings as judged by the investigators. Subjects were also excluded if they had used any prescription drugs, over-the-counter medications (including smoking-cessation medications) or dietary supplements within two weeks prior to the investigational period; or if they had used any tobacco products other than commercial cigarettes (such as heated tobacco products, hand-rolled cigarettes, cigarillos, cigars, pipes, snuff tobacco, chewing tobacco, etc*.*) within a week before screening.

### Sample size determination

A sample size of 15 subjects per study group was set for this study. This was determined based on the expected least squares (LS) mean ratios of the levels of 15 BoE (Table 2), as observed in smokers who switched to heated tobacco products for 5 days in previous studies of similar design^12–16,18^. With the smallest effect size was calculated as 1.60 at *o*-toluidine within 15 BoE, a sample size of 12 subjects in each study group was considered sufficient to attain 80% power to show a reduction of 40% in 15 BoE in the five study groups (four heated tobacco products and SS) compared with the CC group using two-sided tests with 5% alpha level (Bonferroni-correction, α = 0.05/5).

**Table 2:** Biomarkers of exposure to selected harmful and potentially harmful constituents.

Acronym	Biomarker of exposure	HPHC
3-HPMA	3-Hydroxypropyl- mercapturic acid	Acrolein
3-OH-BaP	3-Hydroxy-benzo[*a*]pyrene	Benzo[*a*]pyrene
Total 1-OHP	Total 1-hydroxypyrene	Pyrene^a^
*S*-PMA	*S*-Phenylmercapturic acid	Benzene
MHBMA	Monohydroxybutenyl-mercapturic acid	1,3-Butadiene
eCO^b^	Exhaled carbon monoxide	Carbon monoxide
Total NNAL	Total 4-(methylnitrosamino)-1-(3-pyridyl)-1-butanol	4-(Methylnitrosamino)-1-(3-pyridyl)-1-butanone (NNK)
Total NNN	Total *N*-nitrosonornicotine	*N*-Nitrosonornicotine (NNN)
CEMA	2-Cyanoethylmercapturic acid	Acrylonitrile
4-ABP	4-Aminobiphenyl	4-Aminobiphenyl
1-AN	1-Aminonaphthalene	1-Aminonaphthalene (1-AN)
2-AN	2-Aminonaphthalene	2-Aminonaphthalene (2-AN)
3-HMPMA	3-Hydroxy-1-methylpropylmercapturic acid	Crotonaldehyde
HEMA	2-Hydroxyethyl-mercapturic acid	Ethylene oxide
*o*-Tol	*o*-Toluidine	*o*-Toluidine

^a^Although pyrene is not listed as an HPHC by the FDA, the BoE to pyrene was assessed as an alternative and supportive BoE to benzo[*a*]pyrene^20^.

^b^The exhaled CO level of subjects was measured by using a piCO Smokerlyzer.

### Investigational tobacco products

In this study, four types of heated tobacco products (devices/consumables) available on the Japanese market were used as investigational tobacco products, and each consumable was selected a representative regular flavored one that designed to provide the taste of tobacco. All heated tobacco products were acquired from the market and provided to the clinic free of charge by the sponsor whilst CC were purchased and brought to the clinic by the subjects themselves. The chemical analysis of aerosols generated from each heated tobacco products showed that most of the measured selected cigarette smoke constituents were below quantifiable levels or was lower than that in the 1R6F^19^. Each randomized subject received an instruction on how to use their assigned product before beginning to use it on Day 1.

IT2.0a (device/cartridge and tobacco capsule: Ploom TECH+/MEVIUS Mild Blend for Ploom TECH+) developed by Japan Tobacco Inc., is composed of a puff activated electrical heating device with a rechargeable battery, a cartridge containing nicotine free liquid, and a tobacco capsule containing tobacco granules (Fig. 1a). The IT2.0a generates a thermally vaporized aerosol which then passes through the tobacco capsule (the temperature inside the tobacco capsule during use is about 40 °C) before being inhaled. In doing so, evaporated tobacco-derived flavors and nicotine are infused into the vapor. Aerosol emissions for the tobacco consumables of IT2.0a used in this study are published on our website^21^.

DT2.2a (device/tobacco stick: Ploom S 2.0/MEVIUS Regular Taste for Ploom S) also developed by Japan Tobacco Inc., consists of a rechargeable holding device and a specially designed tobacco stick (Fig. 1b). The tobacco stick consists of flavors added to a tobacco blend covered with paper and a filter on the tip and has a shape resembling a cigarette. The heater in the device heats the tobacco stick (the temperature inside the tobacco stick during use is about 200 °C) to generate tobacco vapor before being inhaled. The DT2.2a has two heating modes, and the “TASTE ACCEL” mode, which lengthens the duration of the peak heating temperature, is designed as the standard mode of this device. Subjects were instructed to use the DT2.2a in the “TASTE ACCEL” mode. Aerosol emissions for the tobacco consumables of DT2.2a used in this study are published on our website^22^.

As major in-market heated tobacco products, THP (device/tobacco stick: glo pro/Kent Neo Sticks Bright Tobacco, manufactured by British American Tobacco) and THS (device/tobacco stick: IQOS 3 MULTI/Marlboro Heat Stick Regular, manufactured by Philip Morris International) were also assessed in the present study. The THP comprises a battery-powered device that heats specially designed tobacco sticks to approximately 240 °C. The THP has two heating modes, one of which, the “Boost Mode,” can shorten preheating time for use, but the subjects were instructed to use THP in the “Standard Mode.” The THS comprises an electronic device that heats specially designed tobacco sticks to approximately 300 °C (up to 350 °C) via a heating blade. Aerosol emissions of both THP and THS have been published previously^8,9,23,24^.

### Assessments

The baseline characteristics of subjects, including gender, age, BMI, smoking history, the tar value of the subject’s usual brand of CC (value printed on each cigarette package), daily cigarette consumption and score on the Fagerström Test for Nicotine Dependence (FTND)^25^, were all recorded at screening.

As shown in Table 2, the study examined the following 15 BoE (3-hydroxypropyl-mercapturic acid [3-HPMA], 3-hydroxy-benzo[a]pyrene [3-OH-BaP], total 1-hydroxypyrene [1-OHP], *S*-phenylmercapturic acid [*S*-PMA], monohydroxybutenyl-mercapturic acid [MHBMA], exhaled carbon monoxide [eCO], total 4-(methylnitrosamino)-1-(3-pyridyl)-1-butanol [NNAL], total *N*-nitrosonornicotine [NNN], 2-cyanoethylmercapturic acid [CEMA], 4-Aminobiphenyl [4-ABP], 1-aminonaphthalene [1-AN], 2-aminonaphthalene [2-AN], 3-hydroxy-1-methylpropylmercapturic acid [3-HMPMA], 2-hydroxyethyl-mercapturic acid [HEMA], *o*-toluidine [*o*-Tol]). These BoE were selected as reliable surrogate markers of exposure to HPHCs and pyrene. They have been widely measured in clinical studies to evaluate exposure from tobacco product use^26–29^. The eCO level of subjects was measured using a piCO Smokerlyzer (Bedfont Scientific Ltd, Maidstone., England) at the same time each evening (between 5:00 p.m. and 7:00 p.m.) on Day − 1, Day 3 and Day 5. Each eCO measurement took place between 30 and 45 min after the subject’s previous use of any investigational tobacco product. The 24-h urine samples for measuring 14 BoE (3-HPMA, 3-OH-BaP, total 1-OHP, *S*-PMA, MHBMA, total NNAL, total NNN, CEMA, 4-ABP, 1-AN, 2-AN, 3-HMPMA, HEMA, *o*-Tol) were pooled from the morning on Day − 1, Day 3, and Day 5 to the following morning. Creatinine was also measured in 24-h urine for adjustment of the concentration of all urinary BoE. All bioanalyses were carried out using validated methods at Celerion Laboratories (Lincoln, NE, USA and Zurich, Switzerland). The details of these methods have been published previously^18^.

Nicotine uptake was assessed by nicotine equivalents (the molar sum of nicotine and the five major metabolites) in the same 24-h urine samples used for BoE assessment. The nicotine equivalents have been widely used as a biomarker of nicotine uptake and reported to reflect approximately 90% of the daily nicotine uptake^30^. The subject’s daily product consumption from Day 1 to Day 5 was recorded for all subjects assigned to the CC and heated tobacco product groups. When recording the daily product consumption of CC, DT2.2a, THP or THS, the number of consumed cigarettes or tobacco sticks was counted. Since a single tobacco capsule for IT2.0a can last for approximately 50 puffs depending on puffing duration, consumed tobacco capsules for IT2.0a were counted based on the blinking of the LED that indicates the replacement time for tobacco capsules. Daily product consumption was equivalent to the number of capsules replaced. If a tobacco capsule remained unfinished on Day 5, it was counted as 0.5 of a used capsule.

The subjective effects were assessed using self-reported questionnaires. After the day’s last use of the investigational tobacco product on Day − 1, Day 3 and Day 5, subjects assigned to the CC and heated tobacco product groups answered the modified Cigarette Evaluation Questionnaire (mCEQ)^31,32^. The following mCEQ subscales were evaluated: Smoking Satisfaction (satisfying, tastes good, and enjoyment of smoking); Psychological Reward (calms down, makes more alert, reduces irritability, helps concentration, reduces hunger); Aversion (dizziness, nausea); Enjoyment of Respiratory Tract Sensations (single-item assessment); and Craving Reduction (single-item assessment). Each item was rated on a 7-point Likert scale ranging from 1 (“Not at all”) to 7 (“Extremely”).

Safety outcomes, including an assessment of adverse events (AEs), were recorded. These comprised any abnormal clinical findings from vital signs, clinical laboratory tests, and physical examinations throughout the study period following randomization.

### Statistical analysis

The primary endpoints were changes in 15 BoE levels in all subjects who used the assigned investigational tobacco product at least once and had at least one BoE assessment after post-randomization. The differences in the ln-transformed values of the BoE between study groups (IT2.0a, DT2.2a, THP, THS and SS) and comparator study group (CC) were evaluated using analysis of covariance, with the endpoints observed at the end of exposure (Day 5) by study group and site, with the baseline (Day − 1) value as a covariant (two-sided significance level with 5% alpha level, Bonferroni-correction, α = 0.05/5). The point estimates of the geometric LS mean ratios and 95% confidence intervals (CI) of the ratio were also calculated for the five study groups.

Descriptive statistics are presented to describe the daily product consumption and nicotine equivalents results for each assessment, and percent changes in values were also calculated from baseline and endpoints observed on Day 5 ([endpoint − baseline]/baseline × 100%). For the questionnaire, the descriptive statistics are presented to describe the scores and changes in scores were calculated from baseline and endpoints observed in each subscale. All statistical analyses were performed using Statistical Analysis Software (SAS), version 9.4 (SAS Institute Inc., North Carolina, USA).

## Results

### Study population

Of the 252 participants screened, 93 subjects were enrolled into the study (Supplemental Fig. 1). During the baseline period, three subjects withdrew from the study for personal reasons or were withdrawn due to non-compliance with the randomization process. One subject who was assigned to the THP group withdrew on Day 1 for personal reasons and was excluded from the analysis because the subject did not have any BoE assessment after post-randomization. Thus, 89 subjects completed the study in accordance with the protocol. There was no substantial difference between any of the six groups in terms of age, sex, BMI, daily cigarette consumption, the tar values of the subject’s usual brand of CC, and FTND score at screening (Table 3).

**Table 3:** Demographic characteristics by study group, at baseline.

Variable and statistics	IT2.0a (n = 15)	DT2.2a (n = 15)	THP (n = 14)	THS (n = 15)	SS (n = 15)	CC (n = 15)	Overall (n = 89)
**Age (year)**							
Mean ± SD	27.6 ± 8.4	26.9 ± 8.6	28.8 ± 7.9	26.6 ± 7.4	26.6 ± 9.3	28.4 ± 10.0	27.5 ± 8.4
Range	21–49	21–47	21–49	21–43	21–49	21–47	21–49
**Sex, n (%)**							
Male	11 (73.3)	11 (73.3)	10 (71.4)	11 (73.3)	11 (73.3)	11 (73.3)	65 (73.0)
Female	4 (26.7)	4 (26.7)	4 (28.6)	4 (26.7)	4 (26.7)	4 (26.7)	24 (27.0)
**BMI (kg/m**^**2**^**)**							
Mean ± SD	21.7 ± 2.3	20.9 ± 2.4	22.7 ± 2.6	22.1 ± 2.2	21.3 ± 2.2	21.6 ± 2.7	21.7 ± 2.4
Range	18.0–26.0	16.9–25.3	18.5–27.7	18.6–27.0	18.5–25.9	19.0–26.8	16.9–27.7
**Daily cigarette consumption (cig/day)**							
Mean ± SD	14.3 ± 3.4	16.1 ± 3.0	13.7 ± 3.0	13.9 ± 3.5	14.1 ± 3.1	13.8 ± 2.5	14.3 ± 3.1
Range	11–20	11–20	11–20	11–20	11–20	11–20	11–20
**Tar level of usual brand (mg)**							
Mean ± SD	7.6 ± 2.9	9.4 ± 3.2	8.1 ± 3.7	7.8 ± 4.7	8.8 ± 3.8	7.2 ± 2.7	8.1 ± 3.7
Range	1–12	3–14	3–14	1–14	3–17	3–12	1–17
**FTND total score (–)**							
Mean ± SD	4.1 ± 1.8	4.7 ± 1.3	3.8 ± 1.1	4.0 ± 1.4	4.1 ± 1.8	3.9 ± 1.1	4.1 ± 1.4
Range	1–8	2–7	3–6	2–7	1–7	3–6	1–8

*BMI* body mass index, *CC* combustible cigarettes group, *DT2.2a* direct tobacco heating system platform 2 generation 2 version a group, *FTND* Fagerström Test for Nicotine Dependence, *IT2.0a* indirect tobacco heating system platform 2 generation 0 version a group, *SD* standard deviation, *SS* stop smoking group, *THP* tobacco heating product group, *THS* tobacco heating system group.

### Product consumption and nicotine uptake

Product consumption is provided in Table 4. Subjects in the CC group smoked approximately 12–13 cigarettes per day (%change on Day 5; 4.7%), and it was confirmed that they kept their daily cigarette consumption of CC within ± 10% of their self-reported daily consumption throughout the investigational period. After switching to heated tobacco products, product consumption increased or remained relatively stable depending on the study group (%change on Day 5; IT2.0a: 96.9%, DT2.2a: 18.1%, THP: 20.9%, THS: 22.1%).

**Table 4:** Product consumption in each study group (consumables/day).

Study group	Day 1	Day 2	Day 3	Day 4	Day 5	%Change from Day 1 on Day 5
	Mean (SD)	Mean (SD)	Mean (SD)	Mean (SD)	Mean (SD)	Mean (SD)
IT2.0a (n = 15)	2.5 (1.3)	3.0 (0.9)	3.7 (1.3)	3.5 (1.8)	4.2 (1.6)	96.9 (99.1)
DT2.2a (n = 15)	11.9 (3.6)	11.6 (3.8)	13.5 (3.6)	12.5 (3.1)	13.3 (3.2)	18.1 (30.5)
THP (n = 14)	11.0 (3.7)	11.0 (3.4)	11.6 (3.3)	10.8 (2.8)	11.9 (2.6)	20.9 (48.8)
THS (n = 15)	10.7 (4.6)	10.9 (4.8)	12.1 (4.8)	11.5 (4.3)	12.3 (4.3)	22.1 (31.1)
CC (n = 15)	12.5 (2.6)	12.7 (2.4)	13.0 (2.5)	12.5 (2.4)	13.0 (2.2)	4.7 (9.5)

*CC* combustible cigarettes group, *DT2.2a* direct tobacco heating system platform 2 generation 2 version a group, *IT2.0a* indirect tobacco heating system platform 2 generation 0 version a group, *SD* standard deviation, *THP* tobacco heating product group, *THS* tobacco heating system group.

The nicotine uptake, estimated by urinary nicotine equivalents, is provided in Table 5. The nicotine uptake was generally stable in the CC group between baseline and the end of the investigational period (%change on Day 5; 15.7%). The nicotine uptake decreased in the SS group from baseline on Day 5 (%change on Day 5; − 94.0%). The nicotine uptake in the THS group was generally stable (%change on Day 5; 3.0%), and the nicotine uptake in the other heated tobacco product groups decreased from baseline on Day 5 (%change on Day 5; IT2.0a: − 57.3%, DT2.2a: − 47.4%, THP: − 14.3%).

**Table 5:** Nicotine uptake in each study group (nicotine equivalents, µg/mg creatinine).

Study group	Baseline	Day 3	Day 5	%Change from baseline on Day 5
	Mean (SD)	Mean (SD)	Mean (SD)	Mean (SD)
IT2.0a (n = 15)	5.31 (1.88)	2.08 (0.58)	2.00 (0.68)	− 57.3 (21.3)
DT2.2a (n = 15)	8.09 (3.33)	3.98 (1.38)	3.99 (1.39)	− 47.4 (14.8)
THP (n = 14)	6.00 (2.75)	4.59 (2.15)	5.02 (2.28)	− 14.3 (16.8)
THS (n = 15)	6.15 (4.31)	5.06 (2.56)	5.38 (2.38)	3.0 (35.3)
SS (n = 15)	6.72 (3.91)	0.79 (0.45)	0.33 (0.13)	− 94.0 (2.5)
CC (n = 15)	5.25 (1.84)	5.57 (2.00)	5.94 (1.92)	15.7 (20.1)

*CC* combustible cigarettes group, *DT2.2a* direct tobacco heating system platform 2 generation 2 version a group, *IT2.0a* indirect tobacco heating system platform 2 generation 0 version a group, *SD* standard deviation, *SS* stop smoking group, *THP* tobacco heating product group, *THS* tobacco heating system group.

### Biomarkers of exposure

**Figure 2:**
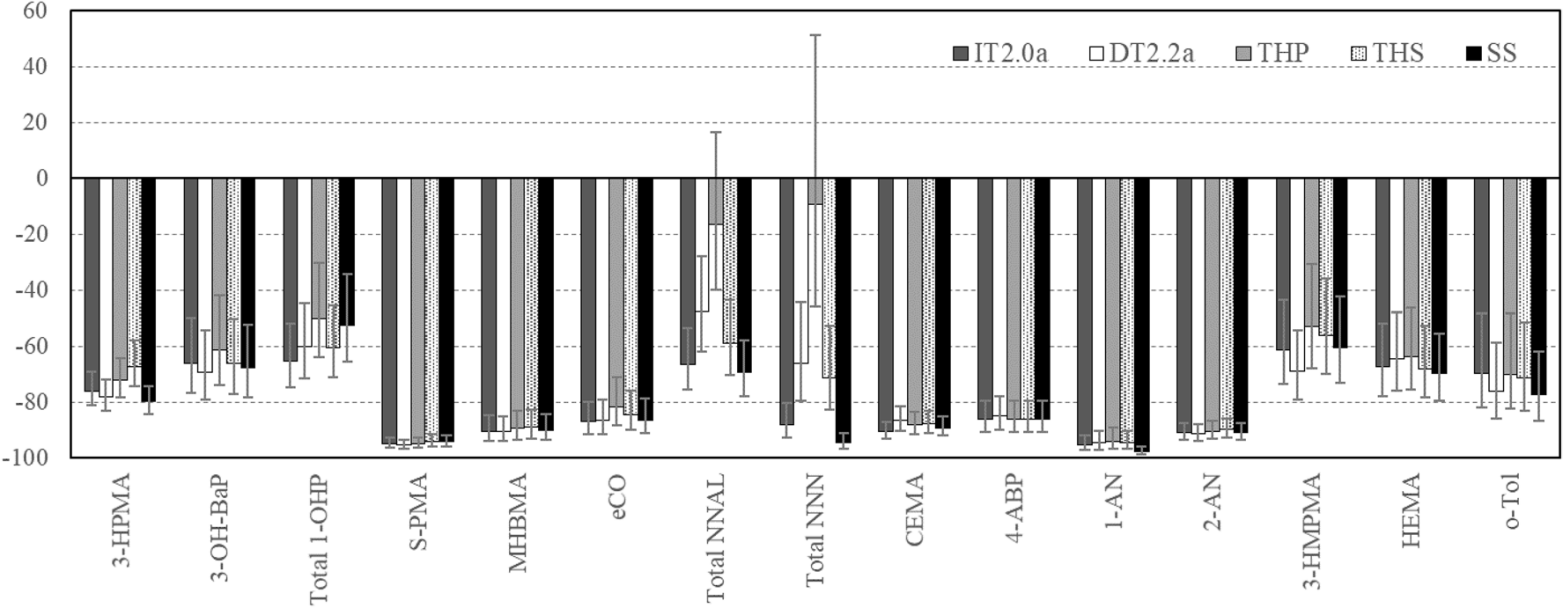
Biomarkers of exposure level reductions on Day 5, IT2.0a, DT2.2a, THP, THS and SS relative to CC (%). Percent reduction values are geometric least squares mean ratio (%) and 95% confidence intervals from analysis of covariance model conducted on ln-transformed Day 5 values with ln-transformed baseline value, study group and site as fixed effect factors on Day 5. *1-OHP* 1-hydroxypyrene, *1-AN* 1-aminonaphthalene, *2-AN* 2-aminonaphthalene, *3-HMPMA* 3-hydroxy-1-methylpropylmercapturic acid, *3-HPMA* 3-hydroxypropyl-mercapturic acid, *3-OHBaP* 3-hydroxy-benzo[a]pyrene, *4-ABP* 4-aminobiphenyl, *CC* combustible cigarettes smoking group, *CEMA* 2-cyanoethylmercapturic acid, *DT2.2a* direct tobacco heating system platform 2 generation 2 version a use group, *eCO* exhaled carbon monoxide, *HEMA* 2-hydroxyethylmercapturic acid, *IT2.0* indirect tobacco heating system platform 2 use group, *NNAL* 4-(methylnitrosamino)-1- (3-pyridyl)-1-butanol, *MHBMA* monohydroxybutenyl-mercapturic acid, *NNN* *N*-nitrosonornicotine, *o-Tol* *o*-toluidine, *S-PMA* *S*-phenylmercapturic acid, *SS* stop smoking group, *THP* tobacco heating product use group, *THS* tobacco heating system use group.

Reductions in BoE levels on Day 5, derived from the geometric LS mean ratio, were observed for all BoE levels to 14 HPHCs and pyrene in the IT2.0a, DT2.2a, THP, THS, and SS groups relative to the CC group (Fig. 2), with statistical data presented in Supplemental Table 1. There were statistically significant differences between the SS group and the CC group for all BoE levels. Except for total NNAL and total NNN in the THP group, a significant reduction in all BoE was observed on Day 5 in those who switched to heated tobacco products compared with the CC group.

The reductions (%) relative to the CC group on Day 5 were of a similar magnitude between the heated tobacco product and SS groups for each BoE except for total NNAL and total NNN. For total NNAL and total NNN in the IT2.0a (− 66.4% and − 88.0%), DT2.2a (− 47.7% and − 66.2%) and THS groups (− 59.0% and − 71.5%), the reductions relative to the CC group were similar or slightly less than those in the SS group (− 69.4% and − 94.6%). For total NNAL and total NNN in the THP group (− 16.4% and − 9.5%), the reductions relative to the CC group were slight compared to the SS group.

Levels of BoE to 14 HPHCs and pyrene at baseline, Day 3, and Day 5 for the IT2.0a, DT2.2a, THP, THS, SS, CC groups are provided in Supplemental Table 2. From baseline to Day 5, an apparent decrease in all BoE levels was commonly observed in the IT2.0a, DT2.2a, THP, THS and SS groups.

### Subjective effects

Table 6 shows five mCEQ subscale scores and the baseline scores were comparable for the five study groups. Except for subscale “Aversion” in the THS and CC groups, all groups reported a fall in mCEQ subscale scores on Day 5 compared to baseline.

**Table 6:** mCEQ subscale scores in each study group.

Subscale of mCEQ*	Baseline	Day 3	Day 5	Change from baseline on Day 5
Study group	Mean (SD)	Mean (SD)	Mean (SD)	Mean (95% CI)
**Smoking satisfaction**				
IT2.0a (n = 15)	4.87 (0.96)	3.18 (0.96)	3.33 (1.05)	− 1.53 (− 2.22, − 0.84)
DT2.2a (n = 15)	4.64 (1.59)	2.49 (0.65)	3.22 (0.96)	− 1.42 (− 2.59, − 0.26)
THP (n = 14)	4.64 (1.56)	2.76 (1.26)	2.95 (1.27)	− 1.69 (− 2.64, − 0.74)
THS (n = 15)	4.09 (0.78)	2.76 (0.89)	2.87 (1.05)	− 1.22 (− 1.82, − 0.62)
CC (n = 15)	4.80 (1.32)	4.67 (1.31)	4.56 (1.59)	− 0.24 (− 0.83, 0.34)
**Psychological reward**				
IT2.0a (n = 15)	3.64 (1.08)	2.80 (0.87)	2.67 (0.80)	− 0.97 (− 1.48, − 0.47)
DT2.2a (n = 15)	3.64 (1.25)	2.35 (0.98)	2.76 (1.19)	− 0.88 (− 1.74, − 0.02)
THP (n = 14)	3.99 (1.37)	2.70 (1.46)	2.63 (1.30)	− 1.36 (− 1.88, − 0.84)
THS (n = 15)	3.40 (1.12)	2.36 (0.87)	2.49 (0.90)	− 0.91 (− 1.37, − 0.44)
CC (n = 15)	3.52 (1.32)	3.65 (1.29)	3.17 (1.38)	− 0.35 (− 0.70, 0.00)
**Aversion**				
IT2.0a (n = 15)	1.40 (0.71)	1.17 (0.36)	1.03 (0.13)	− 0.37 (− 0.74, 0.00)
DT2.2a (n = 15)	1.63 (0.90)	1.03 (0.13)	1.17 (0.52)	− 0.47 (− 1.04, 0.11)
THP (n = 14)	1.54 (0.93)	1.18 (0.25)	1.11 (0.29)	− 0.43 (− 0.98, 0.12)
THS (n = 15)	1.30 (0.49)	1.33 (0.65)	1.37 (0.61)	0.07 (− 0.26, 0.40)
CC (n = 15)	1.37 (0.52)	1.33 (0.56)	1.40 (0.85)	0.03 (− 0.31, 0.37)
**Enjoyment of respiratory tract sensations**				
IT2.0a (n = 15)	4.73 (0.80)	2.73 (1.49)	2.80 (1.08)	− 1.93 (− 2.67, − 1.19)
DT2.2a (n = 15)	4.47 (1.81)	2.53 (1.51)	2.73 (0.96)	− 1.73 (− 2.94, − 0.52)
THP (n = 14)	4.29 (1.68)	2.14 (1.03)	2.64 (1.45)	− 1.64 (− 2.67, − 0.61)
THS (n = 15)	3.87 (1.30)	2.20 (1.08)	2.67 (1.29)	− 1.20 (− 1.83, − 0.57)
CC (n = 15)	4.73 (1.33)	4.40 (1.35)	4.13 (1.64)	− 0.60 (− 1.15, − 0.05)
**Craving reduction**				
IT2.0a (n = 15)	3.87 (1.51)	2.93 (1.16)	3.13 (1.25)	− 0.73 (− 1.85, 0.38)
DT2.2a (n = 15)	4.27 (1.87)	2.80 (1.15)	3.07 (1.28)	− 1.20 (− 1.80, − 0.60)
THP (n = 14)	3.57 (1.55)	3.00 (1.47)	2.93 (1.21)	− 0.64 (− 1.74, 0.46)
THS (n = 15)	4.07 (1.67)	2.67 (1.11)	2.87 (1.19)	− 1.20 (− 2.12, − 0.28)
CC (n = 15)	4.27 (1.91)	3.80 (1.97)	4.07 (1.44)	− 0.20 (− 0.76, 0.36)

*Smoking satisfaction = average score from 3 questions (“Was smoking satisfying?”, “Did the cigarette taste good?” and “Did you enjoy smoking?”); Psychological reward = average scores from 5 questions (“Did smoking calm you down?”, “Did smoking make you feel more awake?”, “Did smoking make you feel less irritable?”, “Did smoking help you concentrate?”, and “Did smoking reduce your hunger for food?”); Aversion = average score from 2 questions (“Did smoking make you dizzy?” and “Did smoking make you nauseous?”); Enjoyment of respiratory tract sensations = score from a question (“Did you enjoy the sensations in your throat and chest?”); Craving reduction = score from a question (“Did smoking immediately reduce your craving for a cigarette”). Each item was rated on a 7-point Likert scale ranging from 1 (“Not at all”) to 7 (“Extremely”). *CC* combustible cigarettes group, *CI* confidential interval, *DT2.2a* direct tobacco heating system platform 2 generation 2 version a group, *IT2.0a* indirect tobacco heating system platform 2 generation 0 version a group, *mCEQ* modified Cigarette Evaluation Questionnaire, *SD* standard deviation, *THP* tobacco heating product group, *THS* tobacco heating system group.

The mean changes from baseline for the IT2.0a, DT2.2a, THP, and THS groups were greater than those for the CC group, but in most cases the changes were not remarkably different between the heated tobacco product groups and CC group with overlap of the 95% CI. For the subscales of “Smoking satisfaction” and “Enjoyment of respiratory tract sensations,” the changes from baseline in the IT2.0a group (− 1.53; 95% CI − 2.22, − 0.84, and − 1.93; 95% CI − 2.67, − 1.19, respectively) were greater than those in the CC group (− 0.24; 95% CI − 0.83, 0.34, and − 0.60; 95% CI − 1.15, − 0.05, respectively) without overlap of the 95% CI. For the subscale of “Psychological reward,” the change from baseline in the THP group (− 1.36; 95% CI − 1.88, − 0.84) was greater than that in the CC group (− 0.35; 95% CI − 0.70, 0.00) without overlap of the 95% CI.

### Safety

The first participant was screened on August 26, 2020, and the last subject was discharged after all safety assessments on October 31, 2020. There were no AEs reported during the study.

## Discussion

As heated tobacco products are becoming more numerous and diverse, the present study was conducted as a comprehensive evaluation of exposure in healthy adult smokers to selected cigarette smoke constituents following use of four in-market heated tobacco products (IT2.0a, DT2.2a, THP, and THS). The SS group was included to provide a benchmark for the potential reduction in exposure associated with smoking cessation. This study was conducted in a highly controlled and short-term confinement conditions to assess exposure associated with the exclusive use of the assigned investigational tobacco products.

The study showed that switching to the four in-market heated tobacco products for 5 days led to a significant reduction in most of the 15 BoE (3-HPMA, 3-OH-BaP, total 1-OHP, *S*-PMA, MHBMA, eCO, total NNAL, total NNN, CEMA, 4-ABP, 1-AN, 2-AN, 3-HMPMA, HEMA, *o*-Tol) related to the 14 HPHCs and pyrene, compared to the continued smoking of combustible cigarettes. The reductions (%) relative to the CC group for these BoE, except for total NNAL and total NNN, in the four in-market heated tobacco product groups ranged from − 50.0 to − 95.5%, and these magnitudes were similar to those seen in the SS group which ranged from − 52.4 to − 97.7%. These results are generally consistent with the similar clinical studies on other heated tobacco products, and the average of reported reduction relative to the CC group is 50% or more for most of BoE after switching from combustible cigarettes to heated tobacco products for 5 days^12–16,18^.

For total NNN as the BoE of NNN, the IT2.0a, DT2.2a, THS, and SS groups showed remarkable reductions relative to the CC group (− 66.2% to − 94.6%). The THP group showed a small reduction in total NNN relative to the CC group (− 9.5%), and no statistically significant reduction was observed between the THP and CC groups. It is noteworthy that in machine-derived emissions from each heated tobacco product examined in the present study^8,9,19,21–24^, the NNN level from THP is slightly higher than that from other heated tobacco products, and this may provide a potential explanation for the nonsignificant reduction in the total NNN in the present study. The result that the reduction in NNN exposure was not significant is consistent with previous clinical studies on THP^17,19,33^.

For total NNAL as the BoE of NNK, the IT2.0a, DT2.2a, THS, and SS groups showed remarkable reductions relative to the CC group (− 47.4% to − 69.4%). The THP group showed a small reduction in total NNAL relative to the CC group (− 16.4%), and no statistically significant reduction was observed between the THP and CC groups. Despite the NNK level from THP not differing greatly from that for THS in machine-derived emissions^8,9,19,23,24^, a similar clinical study reported that the NNK exposure level from THP is higher than that from THS 5 days after switching^17^. This result is consistent with the present study. On the other hand, a recently published study examining changes in BoE in smokers who switched to using THP for 90 days reported a significant reduction in NNK exposure^33^. The elimination half-life of NNK is known to be 10–18 days^34^, and the short duration of the present study is one of factors that the nonsignificant reduction in the total NNAL following a switch to THP. On the other hand, it is also resulting that significant reductions in total NNAL were observed following a switch to other heated tobacco products, so there may be factors other than study duration. Further studies over a longer period than that this 5-day switching study are required to investigate the overall impact of heated tobacco product use on NNK exposure, and may explain and clarify the actual difference in NNK exposure between different heated tobacco products.

The consumption of products during the study period was relatively stable in the CC, DT2.2a, THP, and THS groups (10.7 to 13.5 cigarettes or sticks per day). Previous clinical studies in smokers who switched to using THP or THS for 5 or 90 days reported that product consumption did not change remarkably from the daily consumption of cigarettes at baseline^12–17,33,35–38^, and these results are generally consistent with those of the present study. In the IT2.0a group, the mean daily product consumption on Day 5 (4.2 capsules per day) was nearly double that recorded on Day 1 (2.5 capsules per day). In a similar clinical study, we conducted on another indirect tobacco heating system (Ploom TECH/NTV) it was also found that the mean daily NTV consumption on Day 5 (6.1 capsules per day) was nearly double that recorded on Day 1 (3.3 capsules per day)^18^. On the other hand, other cross-sectional observation studies in Japan have reported that daily NTV users consumed on average 3.6 or 3 capsules per day^5,39^. The results obtained in the present study suggest that subjects in the IT2.0a group had a temporary increase in daily product consumption but daily product consumption may decrease again after adapting to the product. This increase in product consumption in the IT2.0a group did not lead to an increased nicotine uptake, estimated by urinary nicotine equivalents. In other heated tobacco product groups, product consumption was relatively stable during the study period, and nicotine uptake remained stable or had reduced by the end of the exposure period, compared with baseline.

FDA has recommended acquiring information relating to the abuse liability of tobacco products for a candidate MRTP^3^. It is reported that abuse liability is affected by the contribution and interaction of various factors (e.g., pharmacokinetics of nicotine, taste, and other sensory aspects) and one of the informative elements is the subjective effects of product use^40^. In the heated tobacco product groups, the subjective effects, assessed by mCEQ subscales (Smoking Satisfaction, Psychological Reward, Aversion, Enjoyment of Respiratory Tract Sensations, and Craving Reduction), remained stable or were reduced by the end of the exposure period, compared with those seen at baseline. Lüdicke et al*.* reported that the observed trend following switching to THS from CC was shown reducing the mCEQ scores in the short-term and was toward comparable scores in a longer term^35,37^. They concluded that the change in taste, sensorial experience, nicotine uptake, and ritual following a switching from subjects’ own preferred brand of CC to assigned heated tobacco product are likely reasons for reducing the mCEQ scores in the short-term^4,35,37^. While further studies over a longer period than that this 5-day switching study are required, the result obtained in the present study suggest that the abuse liability of heated tobacco products would not exceed that of combustible cigarettes in the short-term.

No adverse events or other clinical concerns were observed in this study. Some non-serious adverse events were reported in previous clinical studies on the heated tobacco products, but none of the adverse events reported were assessed as being related to heated tobacco product use^12,15,17^. These suggest that there are no short-term safety concerns associated with the use of heated tobacco products, as there are with regular smoking and smoking cessation.

In conclusion, the results obtained in the present study indicate that switching to four in-market heated tobacco products (IT2.0a, DT2.2a, THP, and THS) was associated with significant reductions in exposure to most of the selected HPHCs relative to smoking continuation in Japanese smokers. Significantly, the magnitude of the reduction in exposure to most of the selected HPHCs observed in the heated tobacco product groups was close to that observed in the SS group. The present study had several limitations: the study duration was short; thus, the reduction in NNK exposure could not be fully assessed; some subjects may not have fully adapted to their heated tobacco product in terms of their use behavior, including consumption. In future, any evaluation of the reduction in exposure to HPHCs will require long-term clinical studies that allow subjects to adapt their behavior to heated tobacco product use. Additionally, the substantial reduction of human exposure to the selected tobacco toxicants assessed in our study provide an early insight into the potential for reduced risk. Nevertheless, while an exposure reduction is demonstrated, this solely cannot be extrapolated to any conclusive reduction in disease risk. Moreover, it would be beneficial to evaluate whether reduced HPHC exposure is associated with reduced risk of smoking-related diseases by evaluating candidate biomarkers of potential harm, as well as gather scientific evidence of actual risk reduction, such as long-term epidemiological data.

## Supplementary Information

Supplementary Information.

## Data Availability

The data that support the findings of this study are available from the corresponding author upon reasonable request.
