# Supplementary material for: Assessment of the exposure to selected smoke constituents in adult smokers using in-market heated tobacco products: a randomized, controlled study
Source: Sci Rep. 2022 Oct 28;12:18167. doi: 10.1038/s41598-022-22997-1 (PMC9616951; doi:10.1038/s41598-022-22997-1)
Supplement: Supplementary file 1 — Supplementary Information. [file 41598_2022_22997_MOESM1_ESM.pdf]

Supplemental Table 1. Biomarkers of exposure, ratios of IT2.0, DT2.2a, THP, THS and SS relative to CC.

Biomarkers of exposure	Ratios of HTPs or SS/CC geometric LS mean (95% CI)				
	IT2.0a (n = 15)	DT2.2a (n = 15)	THP (n = 14)	THS (n = 15)	SS (n = 15)
3-HPMA [ng/ mg creatinine]	0.24 (0.19, 0.31) **	0.22 (0.17, 0.28) **	0.28 (0.22, 0.36) **	0.33 (0.26, 0.42) **	0.20 (0.16, 0.26) **
3-OH-BaP [fg/ mg creatinine]	0.34 (0.23, 0.50) **	0.31 (0.21, 0.46) **	0.39 (0.26, 0.58) **	0.34 (0.23, 0.50) **	0.32 (0.22, 0.48) **
Total 1-OHP [pg/ mg creatinine]	0.35 (0.25, 0.48) **	0.40 (0.29, 0.55) **	0.50 (0.36, 0.70) **	0.40 (0.29, 0.55) **	0.48 (0.34, 0.66) **
S-PMA [ng/ mg creatinine]	0.05 (0.04, 0.07) **	0.05 (0.03, 0.06) **	0.05 (0.04, 0.07) **	0.06 (0.04, 0.09) **	0.06 (0.04, 0.08) **
MHBMA [ng/ mg creatinine]	0.10 (0.06, 0.15) **	0.10 (0.06, 0.15) **	0.11 (0.07, 0.17) **	0.11 (0.07, 0.17) **	0.10 (0.06, 0.16) **
eCO [ppm]	0.13 (0.08, 0.20) **	0.13 (0.09, 0.21) **	0.18 (0.12, 0.29) **	0.15 (0.10, 0.24) **	0.14 (0.09, 0.21) **
Total NNAL [pg/ mg creatinine]	0.34 (0.24, 0.46) **	0.52 (0.38, 0.72) **	0.84 (0.60, 1.16)	0.41 (0.30, 0.57) **	0.31 (0.22, 0.42) **
Total NNN [pg/ mg creatinine]	0.12 (0.07, 0.20) **	0.34 (0.20, 0.56) **	0.91 (0.54, 1.51)	0.29 (0.17, 0.47) **	0.05 (0.03, 0.09) **
CEMA [ng/ mg creatinine]	0.09 (0.07, 0.13) **	0.13 (0.10, 0.19) **	0.12 (0.09, 0.16) **	0.12 (0.09, 0.17) **	0.11 (0.08, 0.15) **
4-ABP [pg/ mg creatinine]	0.14 (0.09, 0.21) **	0.15 (0.10, 0.22) **	0.14 (0.09, 0.21) **	0.14 (0.09, 0.21) **	0.14 (0.09, 0.21) **
1-AN [pg/ mg creatinine]	0.05 (0.03, 0.08) **	0.05 (0.03, 0.10) **	0.06 (0.03, 0.11) **	0.06 (0.03, 0.10) **	0.02 (0.01, 0.04) **
2-AN [pg/ mg creatinine]	0.09 (0.07, 0.13) **	0.09 (0.06, 0.12) **	0.09 (0.07, 0.13) **	0.10 (0.07, 0.14) **	0.09 (0.07, 0.13) **
3-HMPMA [ng/ mg creatinine]	0.39 (0.27, 0.56) **	0.31 (0.21, 0.46) **	0.47 (0.32, 0.69) **	0.44 (0.30, 0.64) **	0.39 (0.27, 0.58) **
HEMA [ng/ mg creatinine]	0.33 (0.22, 0.48) **	0.35 (0.24, 0.52) **	0.36 (0.25, 0.54) **	0.32 (0.22, 0.47) **	0.30 (0.21, 0.44) **
o-Tol [pg/ mg creatinine]	0.30 (0.18, 0.52) **	0.24 (0.14, 0.41) **	0.30 (0.18, 0.52) **	0.29 (0.17, 0.49) **	0.23 (0.13, 0.38) **

Geometric least squares mean ratio and 95% confidence intervals from analysis of covariance model conducted on ln-transformed Day 5 values with ln-transformed baseline value, group, and site as fixed effect factors on Day 5.

Abbreviations; 1-OHP = 1-hydroxypyrene; 1-AN = 1-aminonaphthalene; 2-AN = 2-aminonaphthalene; 3-HMPMA = 3-hydroxy-1-methylpropylmercapturic acid; 3-HPMA = 3-hydroxypropyl-mercapturic acid; 3-OHBaP = 3-hydroxy-benzo[a]pyrene; 4-ABP = 4-aminobiphenyl; CC = combustible cigarettes smoking group; CEMA = 2-cyanoethylmercapturic acid; DT2.2a = direct tobacco heating system platform 2 generation 2 version a use group; eCO = exhaled carbon monoxide; HEMA = 2-hydroxyethylmercapturic acid; IT2.0 = indirect tobacco heating system platform 2 use group; NNAL = 4-(methylnitrosamino)-1- (3-pyridyl)-1-butanol; MHBMA = monohydroxybutenyl-mercapturic acid; NNN = N-nitrosonornicotine; *o*-Tol = *o*-toluidine; *S*-PMA = *S*-phenylmercapturic acid; SS = stop smoking group; THP = tobacco heating product use group; THS = tobacco heating system use group.

** Statistically significant difference in biomarkers on Day 5 between IT2.0, DT2.2a, THP, THS, and SS groups and CC group (P-values < .001).

Supplemental Table 2. Biomarkers of exposure levels and changes from baseline.

Biomarkers of exposure<br>Study groups	Baseline<br>Mean (SD)	Day3<br>Mean (SD)	Day5<br>Mean (SD)	Difference: Day5 − Baseline<br>Mean (SD)	 (95% CI)
**3-HPMA (ng/ mg creatinine)**					
IT2.0a (n = 15)	619.9 (194.5)	223.0 (136.7)	155.7 (81.4)	−464.2 (165.2)	−555.7, −372.7
DT2.2a (n = 15)	921.2 (368.5)	219.4 (77.7)	161.5 (52.3)	−759.8 (354.8)	−956.2, −563.3
THP (n = 14)	777.4 (292.6)	249.7 (85.9)	185.9 (52.0)	−591.5 (248.5)	−734.9, −448.0
THS (n = 15)	737.4 (588.6)	257.2 (108.8)	207.9 (81.6)	−529.4 (519.5)	−817.1, −241.8
SS (n = 15)	827.3 (459.9)	195.6 (55.7)	136.0 (38.0)	−691.3 (445.7)	−938.1, −444.5
CC (n = 15)	682.3 (245.4)	669.7 (219.6)	646.7 (214)	−35.5 (110.4)	−96.7, 25.6
**3-OH-BaP (fg/ mg creatinine)**					
IT2.0a (n = 15)	83.4 (58.5)	24.2 (13.9)	19.8 (5.8)	−63.6 (59.1)	−96.4, −30.9
DT2.2a (n = 15)	127.4 (108.2)	32.5 (25.6)	20.7 (8.8)	−106.8 (104.8)	−164.8, −48.7
THP (n = 14)	103.1 (48.4)	32.9 (14.7)	25.3 (11.0)	−77.8 (41.3)	−101.6, −53.9
THS (n = 15)	94.8 (60.4)	20.3 (8.1)	20.7 (9.0)	−74.2 (61.7)	−108.4, −40.0
SS (n = 15)	130.7 (88.4)	26.7 (16.3)	21.3 (8.2)	−109.4 (85.6)	−156.8, −62.0
CC (n = 15)	70.5 (48.0)	71.1 (44.3)	66.7 (48.0)	−3.8 (19.2)	−14.4, 6.9
**Total 1-OHP (pg/ mg creatinine)**					
IT2.0a (n = 15)	108.0 (55.6)	33.5 (21.0)	59.7 (29.3)	−48.3 (44.6)	−73.0, −23.6
DT2.2a (n = 15)	164.6 (104.6)	45.5 (22.4)	82.8 (38.2)	−81.8 (87.1)	−130.1, −33.6
THP (n = 14)	122.2 (44.2)	50.9 (18.1)	85.2 (31.0)	−37.0 (37.6)	−58.7, −15.3
THS (n = 15)	120.5 (78.4)	36.3 (19.6)	69.7 (33.6)	−50.8 (74.8)	−92.2, −9.4
SS (n = 15)	149.4 (109.8)	34.9 (19.8)	92.4 (56.0)	−56.9 (105.9)	−115.6, 1.7
CC (n = 15)	107.1 (51.1)	108.6 (41.3)	157.9 (47.8)	50.8 (28.2)	35.2, 66.4
***S*-PMA (ng/ mg creatinine)**					
IT2.0a (n = 15)	1.29 (0.95)	0.10 (0.04)	0.07 (0.03)	−1.22 (0.94)	−1.74, −0.70
DT2.2a (n = 15)	1.67 (1.32)	0.10 (0.05)	0.07 (0.03)	−1.60 (1.31)	−2.33, −0.88
THP (n = 14)	1.26 (0.98)	0.10 (0.06)	0.07 (0.03)	−1.18 (0.96)	−1.74, −0.63
THS (n = 15)	1.19 (0.81)	0.11 (0.04)	0.08 (0.03)	−1.10 (0.81)	−1.55, −0.66
SS (n = 15)	2.01 (1.67)	0.13 (0.07)	0.09 (0.03)	−1.92 (1.65)	−2.83, −1.01
CC (n = 15)	1.57 (1.09)	1.56 (0.88)	1.75 (1.05)	0.174 (0.44)	0.07, 0.42
**MHBMA (ng/ mg creatinine)**					
IT2.0a (n = 15)	1.07 (0.85)	0.09 (0.04)	0.08 (0.02)	−0.99 (0.84)	−1.46, −0.52
DT2.2a (n = 15)	1.22 (1.09)	0.15 (0.16)	0.08 (0.04)	−1.13 (1.09)	−1.74, −0.53
THP (n = 14)	0.88 (0.89)	0.10 (0.04)	0.09 (0.04)	−0.79 (0.88)	−1.30, −0.28
THS (n = 15)	0.87 (0.65)	0.13 (0.15)	0.09 (0.04)	−0.78 (0.66)	−1.14, −0.41
SS (n = 15)	1.17 (1.02)	0.09 (0.03)	0.09 (0.03)	−1.09 (1.02)	−1.65, −0.52
CC (n = 15)	0.96 (0.65)	1.02 (0.66)	1.04 (0.63)	0.07 (0.16)	−0.02, 0.16
**eCO (ppm)**					
IT2.0a (n = 15)	26.3 (7.5)	4.9 (1.6)	3.8 (1.4)	−22.5 (6.6)	−26.2, −18.9
DT2.2a (n = 15)	33.7 (9.8)	4.7 (2.3)	4.3 (1.5)	−29.3 (9.7)	−34.7, −23.9
THP (n = 14)	34.1 (9.2)	6.6 (2.6)	6.0 (2.4)	−28.1 (9.3)	−33.4, −22.7
THS (n = 15)	33.5 (18.4)	6.2 (3.0)	5.4 (3.3)	−28.1 (18.2)	−38.2, −18.1
SS (n = 15)	35.7 (13.2)	6.7 (3.3)	5.2 (3.3)	−30.5 (12.7)	−37.5, −23.4
CC (n = 15)	27.9 (7.8)	30.9 (9.3)	29.0 (8.2)	1.1 (6.3)	−2.4, 4.5
**Total NNAL (pg/ mg creatinine)**					
IT2.0a (n = 15)	49.7 (41.4)	20.6 (15.7)	16.8 (13.0)	−32.9 (31.1)	−50.1, −15.7
DT2.2a (n = 15)	60.4 (34.9)	35.0 (18.0)	32.7 (17.6)	−27.7 (18.5)	−37.9, −17.5
THP (n = 14)	82.9 (51.1)	61.6 (31.0)	65.1 (32.3)	−17.8 (28.7)	−34.4, −1.2
THS (n = 15)	73.7 (99.0)	35.0 (45.9)	32.4 (42.7)	−41.3 (56.4)	−72.5, −10.1
SS (n = 15)	98.3 (108.9)	36.3 (49.5)	31.3 (40.5)	−67.0 (76.3)	−109.3, −24.8
CC (n = 15)	52.1 (40.3)	51.1 (38.6)	56.5 (42.9)	4.4 (12.1)	−2.2, 11.1
**Total NNN (pg/ mg creatinine)**					
IT2.0a (n = 15)	7.92 (12.05)	0.55 (0.39)	0.50 (0.29)	−7.42 (11.91)	−14.01, −0.83
DT2.2a (n = 15)	4.19 (2.76)	1.25 (0.59)	1.19 (0.66)	−3.01 (2.20)	−4.22, −1.79
THP (n = 14)	6.86 (7.68)	5.08 (5.38)	5.04 (5.42)	−1.82 (3.50)	−3.84, 0.21
THS (n = 15)	3.19 (2.46)	0.85 (0.63)	0.92 (0.72)	−2.27 (2.00)	−3.38, −1.16
SS (n = 15)	6.28 (7.52)	0.17 (0.05)	0.17 (0.07)	−6.11 (7.49)	−10.26, −1.97
CC (n = 15)	3.73 (2.75)	3.39 (2.18)	3.46 (2.28)	−0.27 (1.06)	−0.86, 0.32

Supplemental Table 2. (*Continued*)

Biomarkers of exposure<br>Study groups	Baseline<br>Mean (SD)	Day3<br>Mean (SD)	Day5<br>Mean (SD)	Difference: Day5 − Baseline<br>Mean (SD)	 (95% CI)
**CEMA (ng/ mg creatinine)**					
IT2.0a (n = 15)	60.6 (26.3)	7.3 (4.3)	6.5 (4.0)	−54.1 (23.3)	−67.0, −41.2
DT2.2a (n = 15)	89.4 (35.8)	12.8 (5.1)	11.7 (4.7)	−77.7 (32.1)	−95.5, −60.0
THP (n = 14)	80.1 (34.1)	10.7 (6.5)	9.8 (5.3)	−70.3 (31.6)	−88.5, −52.1
THS (n = 15)	77.0 (73.0)	10.1 (8.5)	9.5 (7.9)	−67.5 (65.3)	−103.6, −31.3
SS (n = 15)	89.5 (55.0)	9.6 (5.7)	9.2 (4.9)	−80.3 (50.9)	−108.5, −52.1
CC (n = 15)	71.0 (34.4)	66.3 (28.1)	70.5 (27.1)	−0.6 (13.2)	−7.9, 6.7
**4-ABP (pg/ mg creatinine)**					
IT2.0a (n = 15)	7.11 (3.78)	1.00 (0.45)	1.00 (0.33)	−6.11 (3.72)	−8.18, −4.05
DT2.2a (n = 15)	8.75 (4.64)	1.16 (0.67)	1.19 (0.43)	−7.56 (4.59)	−10.11, −5.02
THP (n = 14)	8.49 (3.73)	1.20 (0.68)	1.13 (0.55)	−7.36 (3.62)	−9.45, −5.27
THS (n = 15)	7.90 (8.28)	1.00 (0.73)	1.15 (0.83)	−6.75 (7.63)	−10.97, −2.53
SS (n = 15)	10.04 (7.63)	1.17 (0.76)	1.12 (0.52)	−8.91 (7.21)	−12.91, −4.92
CC (n = 15)	6.93 (3.55)	7.29 (4.13)	7.53 (3.72)	0.61 (1.12)	0.02, 1.23
**1-AN (pg/ mg creatinine)**					
IT2.0a (n = 15)	74.7 (31.5)	2.4 (1.3)	5.4 (5.5)	−69.3 (30.4)	−86.1, −52.5
DT2.2a (n = 15)	109.9 (89.9)	3.1 (1.9)	6.3 (4.4)	−103.6 (88.2)	−152.5, −54.8
THP (n = 14)	97.1 (69.3)	3.2 (1.7)	6.5 (5.6)	−90.5 (70.8)	−131.4, −49.7
THS (n = 15)	73.6 (60.3)	2.4 (1.8)	6.1 (5.8)	−67.5 (59.7)	−100.6, −34.5
SS (n = 15)	98.5 (63.2)	2.7 (1.8)	2.5 (2.6)	−96.0 (63.0)	−130.9, −61.1
CC (n = 15)	71.4 (30.9)	72.3 (29.0)	75.5 (25.9)	4.1 (12.6)	−2.9, 11.1
**2-AN (pg/ mg creatinine)**					
IT2.0a (n = 15)	17.4 (7.6)	1.7 (0.5)	1.6 (0.5)	−15.8 (7.7)	−20.1, −11.5
DT2.2a (n = 15)	22.3 (9.8)	1.7 (0.6)	1.7 (0.7)	−20.7 (9.6)	−26.0, −15.4
THP (n = 14)	19.2 (7.8)	1.8 (0.7)	1.7 (0.7)	−17.4 (7.6)	−21.8, −13.0
THS (n = 15)	16.9 (14.8)	1.8 (1.0)	1.8 (0.9)	−15.0 (14.2)	−22.9, −7.2
SS (n = 15)	22.1 (14.0)	1.9 (0.8)	1.7 (0.7)	−20.4 (13.7)	−28.0, −12.8
CC (n = 15)	15.8 (7.2)	16.2 (6.5)	17.5 (6.3)	1.7 (3.4)	−0.1, 3.6
**3-HMPMA (ng/ mg creatinine)**					
IT2.0a (n = 15)	151.4 (44.6)	38.0 (16.2)	80.3 (57.6)	−71.2 (68.5)	−109.1, −33.3
DT2.2a (n = 15)	228.4 (82.2)	34.9 (8.6)	63.1 (20.0)	−165.3 (80.9)	−210.0, −120.5
THP (n = 14)	201.0 (86.5)	46.8 (18.3)	91.9 (29.7)	−109.1 (79.0)	−154.8, −63.5
THS (n = 15)	183.9 (136.2)	47.5 (19.3)	84.2 (35.6)	−99.7 (127.2)	−170.1, −29.2
SS (n = 15)	211.6 (136.6)	46.9 (17.5)	83.0 (44.9)	−128.7 (141.0)	−206.7, −50.6
CC (n = 15)	152.8 (54.4)	154.7 (50)	182.3 (53.4)	29.5 (32.2)	11.7, 47.3
**HEMA (ng/ mg creatinine)**					
IT2.0a (n = 15)	1.99 (1.86)	0.88 (0.67)	0.68 (0.43)	−1.31 (1.59)	−2.19, −0.43
DT2.2a (n = 15)	3.39 (2.31)	1.23 (0.74)	0.96 (0.54)	−2.43 (1.93)	−3.49, −1.36
THP (n = 14)	3.37 (3.34)	1.03 (0.69)	0.90 (0.61)	−2.47 (2.91)	−4.15, −0.79
THS (n = 15)	2.06 (1.38)	0.84 (0.58)	0.68 (0.46)	−1.39 (1.15)	−2.02, −0.75
SS (n = 15)	3.14 (2.41)	1.11 (1.16)	0.85 (0.83)	−2.29 (2.08)	−3.44, −1.13
CC (n = 15)	2.46 (1.18)	2.63 (1.36)	2.45 (1.31)	−0.01 (0.49)	−0.28, 0.26
***o*-Tol (pg/ mg creatinine)**					
IT2.0a (n = 15)	118.1 (59.3)	39.6 (21.3)	34.6 (20.0)	−83.4 (47.4)	−109.7, −57.1
DT2.2a (n = 15)	194.2 (280.8)	33.6 (33.3)	30.1 (19.9)	−164.1 (265.0)	−310.9, −17.4
THP (n = 14)	117.8 (45.1)	34.7 (34.6)	52.3 (86.6)	−65.5 (104.4)	−125.8, −5.2
THS (n = 15)	110.1 (65.1)	28.3 (30.2)	34.4 (30.2)	−75.7 (53.0)	−105.1, −46.4
SS (n = 15)	107.2 (59.8)	19.2 (6.5)	23.1 (13.3)	−84.1 (56.8)	−115.6, −52.7
CC (n = 15)	93.0 (30.8)	87.1 (26.4)	94.6 (29.0)	1.5 (19.0)	−9.0, 12.1

Abbreviations; 1-OHP = 1-hydroxypyrene; 1-AN = 1-aminonaphthalene; 2-AN = 2-aminonaphthalene; 3-HMPMA = 3-hydroxy-1-methylpropylmercapturic acid; 3-HPMA = 3-hydroxypropyl-mercapturic acid; 3-OHBaP = 3-hydroxy-benzo[a]pyrene; 4-ABP = 4-aminobiphenyl; CC = combustible cigarettes smoking group; CEMA = 2-cyanoethylmercapturic acid; DT2.2a = direct tobacco heating system platform 2 generation 2 version a use group; eCO = exhaled carbon monoxide; HEMA = 2-hydroxyethylmercapturic acid; IT2.0 = indirect tobacco heating system platform 2 use group; NNAL = 4-(methylnitrosamino)-1- (3-pyridyl)-1-butanol; MHBMA = monohydroxybutenyl-mercapturic acid; NNN = N-nitrosonornicotine; o-Tol = *o*-toluidine; SD = standard deviation; S-PMA = *S*-phenylmercapturic acid; SS = stop smoking group; THP = tobacco heating product use group; THS = tobacco heating system use group.

Informed Consent Obtained	N = 252

Randomized	N = 93
IT2.0a	N = 15
DT2.2a	N = 16
THP	N = 16
THS	N = 16
SS	N = 15
CC	N = 15

Excluded	N = 159
Screen failure	N = 65
Sample size sufficient	N = 64
Withdrawal by subject	N = 30

Use Investigational Tobacco Product	N = 90
IT2.0a	N = 15
DT2.2a	N = 15
THP	N = 15
THS	N = 15
SS	N = 15
CC	N = 15

Did not Use Investigational Tobacco Product	N = 3
IT2.0a	N = 0
DT2.2a	N = 1
THP	N = 1
THS	N = 1
SS	N = 0
CC	N = 0

Completed	N = 89
IT2.0a	N = 15
DT2.2a	N = 15
THP	N = 14
THS	N = 15
SS	N = 15
CC	N = 15

Discontinued	N = 1
IT2.0a	N = 0
DT2.2a	N = 0
THP	N = 1
THS	N = 0
SS	N = 0
CC	N = 0

Supplemental Figure 1. Disposition of subjects (Flowchart)

Abbreviations; CC = combustible cigarettes smoking group; DT2.2a = direct tobacco heating system platform 2 generation 2 version a use group; IT2.0 = indirect tobacco heating system platform 2 use group; SS = stop smoking group; THP = tobacco heating product use group; THS = tobacco heating system use group.
